# Using functional near‐infrared spectroscopy to measure prefrontal cortex activity during dual‐task walking and navigated walking: A feasibility study

**DOI:** 10.1002/brb3.2948

**Published:** 2023-03-14

**Authors:** Alexander Kvist, Lucian Bezuidenhout, Hanna Johansson, Franziska Albrecht, Urban Ekman, David Moulaee Conradsson, Erika Franzén

**Affiliations:** ^1^ Department of Neurobiology, Care sciences and Society, Division of Physiotherapy Karolinska Institutet Stockholm Sweden; ^2^ Faculty of Community and Health Sciences University of Western Cape Cape Town South Africa; ^3^ Women's Health and Allied Health Professionals Theme, Medical Unit Occupational Therapy & Physiotherapy Karolinska University Hospital Stockholm Sweden; ^4^ Department of Neurobiology, Care Sciences and Society, Division of Clinical Geriatrics Karolinska Institutet Stockholm Sweden; ^5^ Women's Health and Allied Health Professionals Theme, Medical Unit Medical Psychology Karolinska University Hospital Stockholm Sweden

**Keywords:** dual‐task walking, fNIRS, navigation, walking

## Abstract

**Introduction:**

While functional near‐infrared spectroscopy (fNIRS) can provide insight into motor‐cognitive deficits during ecologically valid gait conditions, the feasibility of using fNIRS during complex walking remains unknown. We tested the process and scientific feasibility of using an fNIRS device to measure cortical activity during complex walking tasks consisting of straight walking and navigated walking under single and dual‐task (DT) conditions.

**Methods:**

Nineteen healthy people from 18 to 64 years (mean age: 45.7 years) participated in this study which consisted of three complex walking protocols: (i) straight walking, DT walking (walking while performing an auditory Stroop task) and single‐task auditory Stroop, (ii) straight and navigated walking, and (iii) navigated walking and navigated DT walking. A rest condition (standing still) was also included in each protocol. Process feasibility outcomes included evaluation of the test procedures and participant experience during and after each protocol. Scientific feasibility outcomes included signal quality measures, and the ability to measure changes in concentration of deoxygenated and oxygenated hemoglobin in the prefrontal cortex.

**Results:**

All participants were able to complete the three protocols with most agreeing that the equipment was comfortable (57.9%) and that the testing duration was adequate (73.7%). Most participants did not feel tired (94.7%) with some experiencing pain (42.1%) during the protocols. The signal qualities were high for each protocol. Compared to the rest condition, there was an increase in oxygenated hemoglobin in the prefrontal cortex when performing dual‐task walking and navigation.

**Conclusion:**

We showed that our experimental setup was feasible for assessing activity in the prefrontal cortex with fNIRS during complex walking. The experimental setup was deemed acceptable and practicable. Signal quality was good during complex walking conditions and findings suggest that the different tasks elicit a differential brain activity, supporting scientific feasibility.

## Graphical abstract

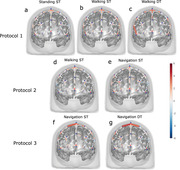

## 1 INTRODUCTION

Performing complex tasks involving a combination of motor (e.g., walking) and cognitive skills forms part of everyday living (Kahya et al., 2019), such as walking while talking or navigating in different environments. In healthy individuals, walking is an automated skill requiring minimal cognitive control which is critical for safe ambulation (Bürki et al., 2017; Clark, 2015), but this control is subject to age‐related changes (Papegaaij et al., 2014). In recent decades, rising evidence has shown the negative effect of aging and neurological diseases (e.g., Parkinson's disease and multiple sclerosis) on motor‐cognitive interaction necessary for an independent lifestyle (Kahya et al., 2019; Roy et al., 2017; Singhal et al., 2020). A growing amount of literature highlights a connection between poor cognitive function, gait variability measures, and falling (Amboni et al., 2013), which bears devastating consequences on health in aging and for people with neurological diseases (Batchelor et al., 2012; Bishnoi et al., 2021). While the neural substrates underlying motor‐cognitive difficulties are not fully understood (McIsaac et al., 2018), one first step is to arrive at a method that accurately portrays these deficits in an ecologically valid state.

Recently, walking automaticity has been assessed with functional Magnetic Resonance Imaging (fMRI) in conjunction with imagined walking or foot movements (Bürki et al., 2017) or by behavioral assessment in studies using a dual‐task paradigm (Johansson et al., 2021; Kressig et al., 2008). Dual‐task (DT) studies involve the simultaneous performance of a primary (e.g., walking) and a secondary task with different goals, the primary task performed alone as a single task (ST) as well as with the secondary task (i.e., DT), for example, walking while performing a cognitive task. The difference in the performance of the primary task under ST and DT conditions provide an indication of the automaticity of the task of interest (Clark, 2015). Although fMRI is considered the gold standard for neuroimaging, the method is sensitive to motion artifacts (Bishnoi et al., 2021; Gramigna et al., 2017) and cannot be used during real walking. Instead, functional near‐infrared spectroscopy (fNIRS) has been introduced as a technique to record cortical activity during gait based on the brain's hemodynamics (Bishnoi et al., 2021). The major advantages of fNIRS compared to other neuroimaging techniques are that it is noninvasive, can be used wirelessly, is lightweight, and relatively robust to head movements (Gramigna et al., 2017; Kahya et al., 2019), which makes it a suitable neuroimaging technique to study complex movements in humans.

Some fNIRS studies investigating DT walking and postural control tasks in healthy adults have found increased oxygenation in the prefrontal cortex (PFC) during DT compared to ST (Holtzer et al., 2011; Marusic et al., 2019; Rosso et al., 2017). Overactivation in cortical areas in older adults has been attributed to compensation for age‐related declines in brain structure and function (Rosso et al., 2017), suggesting that the PFC plays an important role in the performance of motor‐cognitive dual‐tasks.

While fNIRS has been successfully used to measure PFC oxygenation during steady‐state DT walking, to the best of our knowledge, no study has tested the feasibility of using fNIRS during complex walking paradigms combining DT and navigation. Complex walking might pose a challenge to participants and could reduce fNIRS signal quality. Therefore, we evaluated the process and scientific feasibility of measuring PFC activity during complex walking protocols involving both navigation and cognitive DT. Process feasibility aspects included acceptability of wearing the measurement systems, adherence to walking protocols and involved tasks, and practicability of simultaneous fNIRS and gait measures. The scientific feasibility was evaluated by investigating signal quality and differences in the fNIRS data between the different task conditions within the walking protocols.

## 2 MATERIALS AND METHODS

### 2.1 Participants

Nineteen adults (13 females, mean age and range: 45.7, 18–64 years) with the ability to ambulate independently and without any neurological disease or impairment affecting gait were recruited through advertisements. This study was approved by the Regional Board of Ethics in Stockholm (Dnr 2020–05315/2021‐01329). All participants received verbal and written information and gave written consent prior to study participation.

### 2.2 Procedure

Prior to the experimental session, participants were asked about health status and to provide their head size (circumference) for the fNIRS measurement. To describe the demographics as well as the physical and cognitive functioning of the study sample, interviews and a neuropsychological test battery were carried out during the experimental session, in addition to the fNIRS measurement. Demographic data consisted of sex, medical history, years of education, living situation and employment status, as well as overall physical activity level quantified using the Frändin and Grimby scale (Frändin et al., 1991). Participants were also asked to fill in questionnaires concerning: (1) balance confidence using Activities‐specific Balance Confidence (ABC) (Clark, 2015); (2) walking ability using Walk‐12 (Holland et al., 2006); and (3) symptoms of anxiety and depression using the Hospital Anxiety and Depression Scale (HADS) (Zigmond & Snaith, 1983). The neuropsychological test battery comprised the following tests: The Color‐Word Interference Test (CWIT) part III from the Delis‐Kaplan Executive Function System (D‐KEFS) (Delis, 2001), Verbal Fluency part I‐III (from D‐KEFS), Trail Making Test (TMT) part II and IV (from D‐KEFS) and Ray Auditory Verbal Learning Test (RAVLT) (Schmidt, 1996).

### 2.3 Complex walking protocols

**FIGURE 1:**
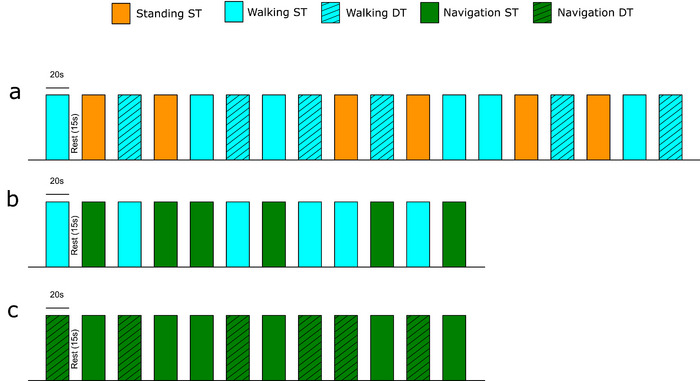
The three complex walking protocols used in the experiment. (a) Walking with a cognitive dual‐task; (b) navigation; and (c) navigation with simultaneous cognitive dual‐task.

The fNIRS experiment consisted of three protocols with different complex walking conditions. The protocols were set up according to a block design, with stimuli being walking conditions and cognitive tasks and rest being standing still. Walking conditions in the first protocol were straight walking (Walking ST), standing still while performing an auditory Stroop task (Standing ST) and straight walking while performing the auditory Stroop task (Walking DT) (Figure 1a). Conditions in the second protocol were Walking ST and navigated walking (Navigation ST) (Figure 1b). Conditions in the third protocol were Navigation ST and navigation while performing the auditory Stroop task (Navigation DT) (Figure 1c).

**FIGURE 2:**
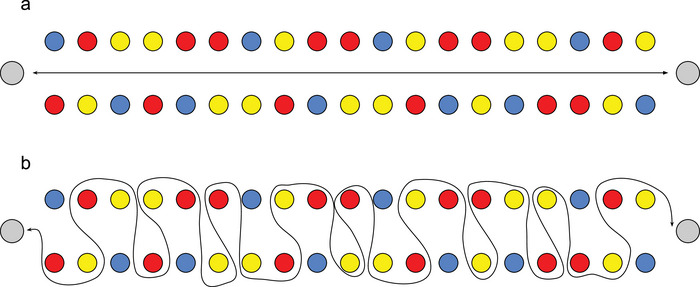
The walking tasks used in the complex walking protocols showing (a) straight walking and (b) navigated walking. In protocols 1 and 3, we combined straight walking and navigated walking with simultaneous performance of the auditory Stroop task. Cones are illustrated in red, yellow, and blue along with cones for turning around in grey. Walking path is illustrated in black.

For the straight walking condition, participants were asked to walk straight at a self‐selected speed to a cone 30 m from the starting cone and back (Figure 2a). For the navigation condition, participants were instructed to walk through a maze consisting of a randomized distribution of 90° to 225° turns to the left and right (Figure 2b). The maze consisted of yellow, red, and blue cones. Participants were instructed to walk around the cones alternating between yellow and red cones and to ignore the blue cones. The auditory Stroop task, which has been proven feasible to perform during gait assessments (Johansson et al., 2021), consisted of the Swedish words for high and low in a congruent or incongruent high and low pitch. Words were presented to the participants through wireless headphones. Participants were instructed to respond verbally, as fast and correctly as possible, to the corresponding pitch irrespective of the words presented. The responses were recorded to analyze task accuracy. During each block with an auditory Stroop task, a total of 7‐word prompts of high or low were presented in a predetermined randomized order. Participants were instructed to pay equal attention to both tasks when dual‐tasking.

Stimulus length was 20 s long, followed by 15 s of rest period to allow for a baseline measure (Amaro & Barker, 2006). Each block condition was performed 6 times in each protocol (e.g., 6 blocks of walking straight) with the time being approximately 12, 8, and 8 min, for protocol 1, protocol 2, and protocol 3, respectively. The conditions within the protocols were randomized to negate the learning effect due to repetition as much as possible.

All test instructions and auditory Stroop were provided through headphones and the test leaders had no interaction with participants during data recording. Participants were given opportunity to practice the auditory Stroop alone and as a DT before starting each protocol. Participants were also given the opportunity to rest for a few minutes in between the protocols.

#### 2.3.1 fNIRS and complex walking measurement

**FIGURE 3:**
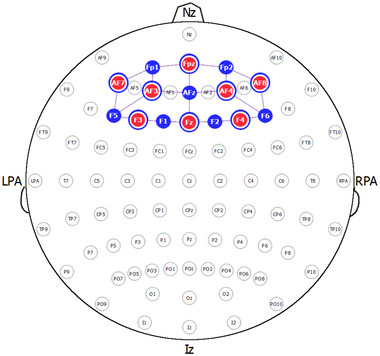
Optode placement according to 10–20 system (created with NirSite 2.0, NIRx Medical Technologies). Blue color indicates detector and red color indicates source. Blue rings around red sources indicate short‐separation detectors. LPA/RPA, left/right preauricular; Iz, inion; Nz, nasion.

The measurement of changes in concentration of oxygenated (HbO) and deoxygenated (HHb) hemoglobin in the PFC was assessed using a NIRSPORT 2 (NIRx Medizintechnik, Berlin, Germany) continuous wave fNIRS device. During testing, the participants were fitted with a headcap consisting of 8 sources and 8 detectors (3 cm separation), and a controller box (approximately 800 g) attached to a backpack harness. The source optodes transmitted light at 760 and 850 nm, and data was sampled at 10 Hz. Optode placement was arranged according to the international 10–20 system over the prefrontal area (Figure 3). To allow for removal of peripheral interference (stemming from blood flow changes in extracerebral layers of the head), short‐separation reference channels (Gagnon et al., 2012) (8 mm separation) were used. The fNIRS optodes were shielded from ambient light by covering the headcap with a black shower cap. The collected fNIRS data was streamed wirelessly to a local computer using Aurora (version 1.4) software. Event triggers delineating block conditions were sent via a lab streaming layer to Aurora. At the same time, gait parameters such as step time and walking speed were collected using three wireless inertial sensors (Opal, APDM Inc.) positioned over the lumbar and on top of each foot near the ankle, although detailed analysis was out of the scope of this feasibility study. To measure mistakes during navigation, a camera (GoPro Hero 8) was mounted on the chest of each participant pointing toward the feet. A navigational mistake was defined as the participant not moving between alternating yellow and red cones (such as yellow to yellow). For the auditory Stroop task, verbal responses were recorded through Audacity (version 2.4.2).

### 2.4 Feasibility assessment

The *process and scientific feasibility* of measuring PFC activity during complex walking with fNIRS were evaluated. Participants were asked questions directly after each protocol regarding their experience of wearing the fNIRS system and their ability to concentrate on the protocol tasks. After completing all protocols, participants filled out a feasibility questionnaire regarding the acceptability of the whole experiment, with questions pertaining to discomfort, dizziness, pain, fatigue, attention, task prioritization and task difficulty (see Appendix A and B for the full questionnaire).

To assess *acceptability* of the experiment, questionnaires were analyzed to evaluate if the experiment was performed in an acceptable timeframe and if participants felt tired, dizzy, or experienced pain during or after the experiment. The ability of participants to concentrate during the experiment was also evaluated. For *adherence* to the walking protocols and involved tasks, completion rates, interruptions during the walking protocols and task performance were evaluated. *Practicability* was assessed by evaluating if data from all measurement systems could be obtained simultaneously and if data about task performance (i.e., audio recordings and video recordings) was sufficient to be able to score the tasks. Assessing *scientific feasibility* involved investigating whether the fNIRS data could reveal changes in PFC activity during protocol tasks and if these differences were in line with previous DT studies, where DT walking is generally associated with an increase in prefrontal activity compared to rest (Vitorio et al., 2017). Signal quality was evaluated by calculating scalp coupling index (SCI) values based on the photoplethysmographic cardiac waveform's presence in the fNIRS signal (Pollonini et al., 2016). SCI values range from 0 to 1, where 1 is considered an ideal coupling value and a threshold for acceptable coupling is around 0.7 to 0.8 (Hernandez et al., 2020; Pollonini et al., 2014).

### 2.5 Data analysis

The fNIRS time series signals were preprocessed and analyzed using the NIRS Brain AnalyzIR toolbox (v2022.4.26) (Santosa et al., 2018). Preprocessing included resampling the raw fNIRS signal to 4 Hz, to account for high autocorrelation in the fNIRS signal, and converting the raw intensity to changes in optical density. Optical density was converted to changes in hemoglobin concentration using the modified Beer‐Lambert law (Delpy et al., 1988). Partial path‐length factor (PPF) was set to 0.1 (Toyoda et al., 2008). Statistical analyses employed a general linear model (GLM). First‐level analysis used an autoregressive model‐based prewhitening filter and robust regression to reduce the effects of physiological and motion induced noise, respectively (Barker et al., 2013; Santosa et al., 2018). Short‐separation channels were added as regressors to the models. For second‐level (group‐level) analysis, a mixed effects model was used where task condition was used as the fixed effect along with a random intercept for each participant. The second‐level model utilized the noise covariance from the first‐level models to solve the mixed effect model using weighted least‐squares regression (Santosa et al., 2018). For contrasting overall activity over the whole PFC between conditions, region of interest (ROI) analyses were performed across long‐separation channels. Finally, *p* values and false‐discovery rate (FDR) corrected *p* values (*q* values) were obtained for comparison between block conditions (Santosa et al., 2018). Significance level was considered as *q* < .05. SCI values were calculated using the MNE‐Python (Gramfort et al., 2013) library (v1.1.0). Descriptive statistics were used to calculate demographic characteristics and results from self‐report questionnaires and feasibility questionnaires, using IBM SPSS (v28.0.1.0). Statistical analysis of auditory Stroop performance was carried out in R(v4.1.1).

## 3 RESULTS

### 3.1 Demographics

Nineteen participants (age range 18–64 years, 68.4% females) participated in this study (Table 1). Most participants reported to be working (84.2%), living with a spouse (63.2%) and being moderately to highly physically active (52.6%, i.e., 5 on the Frändin and Grimby scale).

**TABLE 1:** Demographic and clinical characteristics

Sex, female *n* (%)	13 (68.4)
Age (years), range, mean (SD)	18–64, 45.7 (13.4)
Body mass index (kg/m^2^), range, mean (SD)	20.3–30.1, 24.2 (2.6)
Education (years), range, mean (SD)	12–23, 16.9 (3.1)
Living status	
Alone, *n* (%)	7 (36.8)
With spouse, *n* (%)	12 (63.2)
Occupation	
Working, *n* (%)	16 (84.2)
Student, *n* (%)	2 (10.5)
Unemployed, *n* (%)	1 (5.3)
Falls in last 6 months	
Yes, *n* (%), range	5 (26.3), 1–3
No, *n* (%)	14 (73.7)
Hospital Anxiety and Depression Scale	
Anxiety, 0–21, range, mean (SD)	0–15, 4.8 (3.6)
Depression, 0–21, range, mean (SD)	0–6, 2.2 (1.6)
Frändin and Grimby (1–6)	
3, *n* (%)	6 (31.6)
4, *n* (%)	1 (5.3)
5, *n* (%)	10 (52.6)
6, *n* (%)	2 (10.5)

### 3.2 Process feasibility adherence to walking protocols and involved tasks

All participants were able to complete the three walking protocols successfully without any interruptions, except one participant who had to restart protocol 2 because the wireless headphones turned off.

#### 3.2.1 Protocol 1 (Standing ST and Walking ST/DT)

A majority of the participants did not perceive the Standing ST (90%) and the Walking DT (85%) to be challenging. During dual‐tasking, 55% of the participants reported that they were equally focused on both tasks while 40% stated that they were more focused on the auditory Stroop task. Almost all participants (*n* = 17) scored 100% correct on the auditory Stroop (range 89%−100%).

#### 3.2.2 Protocol 2 (Walking ST and Navigation ST) and Protocol 3 (Navigation ST/DT)

None of the participants perceived that the Navigation ST was challenging while half of the participants reported that the Navigation DT was challenging. During dual‐tasking, most of the participants (65%) felt that they focused on both tasks equally, with 90% of the participants reporting that they perceived both tasks to be of equal importance. Most participants (*n* = 14) responded 100% correctly on the auditory Stroop (range 95%−100%). Visual inspection of navigation indicated that participants understood the task and could perform the navigation with high compliance.

### 3.3 Acceptability of wearing the measurement systems

A majority of participants reported that the fNIRS device was comfortable to wear (57.9%). While most participants did not experience any pain during or after testing (57.9%), some did experience pain during testing (42.1%) and of these, some also after testing (10%). Most pain was reported as some form of pressure on the forehead, while two participants reported pain on the head in general. Wearing the tight fNIRS cap while walking did not make the participants dizzy during (95%) or after (95%) the walking protocols. Participants reported that testing time was adequate (73.7%) and that they did not feel tired during (94.7%) or after (78.9%) testing. All participants (100%) reported that they were able to concentrate during the testing. From putting on equipment to completing the last protocol, the fNIRS measurement took approximately 45 min.

### 3.4 Practicability of simultaneous measurement

Accelerometer data from the APDM system could successfully be obtained for each walking protocol. GoPro videos of each walking protocol were successfully obtained, and cones were sufficiently visible for scoring. Manually synchronizing the start of audio instructions and start of data recording of each system was possible.

### 3.5 Scientific feasibility

Average SCI values calculated for each condition were good, around 0.96, throughout each condition (Table 2). Participants generally had few channels with poor coupling.

**TABLE 2:** Average SCI values over all source and detector pairs (excluding short‐separation channels) for all participants for different protocol conditions, and average number of channels with poor coupling (SCI < 0.7) per participant

*Protocol 1*	SCI, mean (SD)	Poor channels per participant, range, median (IQR)
Standing ST (standing still while preforming AS)	0.961 (0.087)	0–5, 0 (1.5)
Walking ST (straight walking)	0.964 (0.069)	0–4, 0 (0.0)
Walking DT (straight walking while preforming AS)	0.962 (0.071)	0–4, 0 (0.0)
*Protocol 2*		
Walking ST (straight walking)	0.966 (0.080)	0–4, 0 (0.5)
Navigation ST (navigated walking)	0.964 (0.080)	0–3, 0 (0.5)
*Protocol 3*		
Navigation ST (navigated walking)	0.964 (0.064)	0–3, 0 (0.5)
Navigation DT (navigated walking while performing AS)	0.964 (0.061)	0–2, 0 (0.0)

Abbreviations: AS, auditory Stroop; IQR, interquartile range.

**FIGURE 4:**
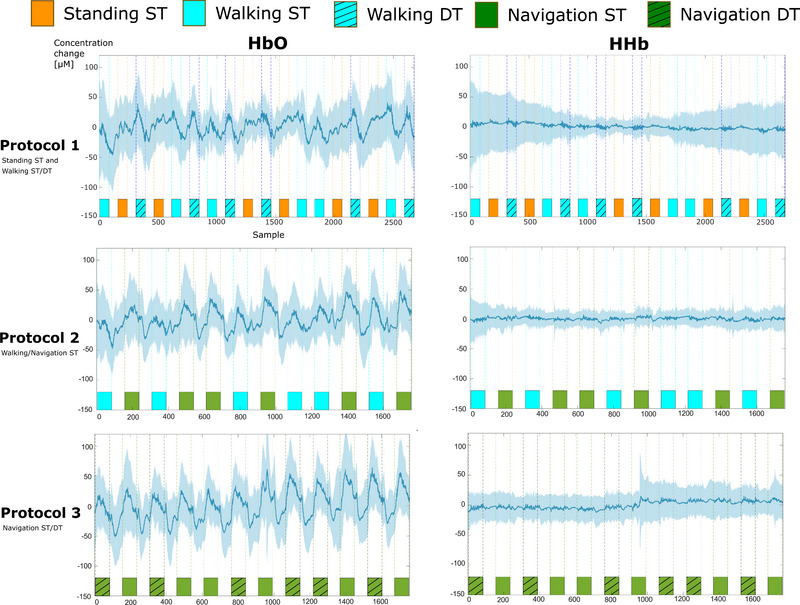
Average time series of HbO and HHb changes for the different complex walking protocols for two channels (source Fz to detectors F1 and F2) across all participants. Stimulus blocks are underlaid on the bottom of each plot. The shaded area represents one standard deviation in each direction.

Figure 4 shows the average time series of recorded fNIRS data in terms of HbO and HHb for the different complex walking protocols for two channels (Fz‐F1 and Fz‐F2), averaged across all participants. The HbO data showed larger amplitudes than the HHb data and generally increased during stimulus blocks compared to rest.

**FIGURE 5:**
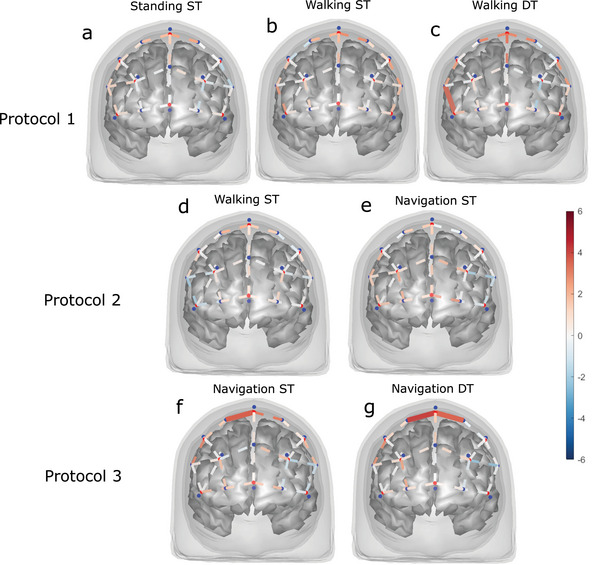
Group‐level analysis of the changes in HbO for (a) Standing ST, (b) Walking ST, (c) Walking DT, (d) Walking ST, (e) Navigation ST, (f) Navigation ST, and (g) Navigation DT. The figures show the *t*‐statistics of each channel, with significantly active channels (*q* < .05) being solid lines. The blue to red color bar denotes the *t*‐statistic value, which indicates an increase or decrease in HbO concentration from baseline to the task condition. One participant was not included in any protocol due to issues with block separation triggers, and another participant was excluded for the same reason in protocol 3.

Figure 5 shows the results of the second‐level (group‐level) analysis, illustrating the mean changes in HbO concentration in the PFC for the different protocols and task conditions. There was a significant increase in activity for Walking DT compared to baseline in the PFC (1 channel, AF8‐F6, β = 2.74, *t*(48) = 3.59, *q* = .039; Figure 5c) whereas no differences were observed for Walking ST (Figure 5b) and Standing ST (Figure 5a) in protocol 1. Neither Navigation ST (Figure 5e) nor Walking ST (Figure 5d) elicited a significant increase from baseline in protocol 2. Protocol 3 showed relatively similar activation for both conditions, with the Navigation ST (Figure 5f) having one channel with a significant increase from baseline (1 channel, Fz‐F2, β = 5.03, t(32) = 3.67, *q* = .043) and the Navigation DT (Figure 5g) having two channels with a significant increase (2 channels, Fz‐F1 and Fz‐F2, β = 5.08, *t*(32) = 3.53, *q* = .043 and *β* = 5.70, *t*(32) = 4.13, *q* = .024).

ROI analysis of condition contrasts (Appendix C.3) showed a trend of increase in HbO and decrease in HHb for DT against ST except in protocol 3, and navigation against straight walking, further enhanced by the difference between HbO and HHb.

## 4 DISCUSSION

This study explored the feasibility of using fNIRS to observe changes in the PFC while performing three ecologically valid complex walking protocols in healthy adults. A feasibility questionnaire of each participant's experience during each protocol showed that participants did not report any major acceptability issues wearing the fNIRS device or performing the experiment. Analysis of performed tasks showed that participants were able to complete the protocols without problems and with high task performance. Practicability could be increased by automatic synchronization of measurement systems. Scientific feasibility was evaluated via signal quality, which was high, and by exploring the activation in the PFC during the different conditions in the three protocols, where a group‐level analysis of the fNIRS data showed activation in the PFC when performing DT walking and navigation compared to rest.

The present results are consistent with previous studies such as Nieuwhof et al. (2016) who showed that participants experienced a low burden of wearing a wireless fNIRS device during DT walking, and were able to perform walking protocols with little effort. Although some participants reported experiencing pain in our experiment, this was generally reported as pressure from the optodes on the forehead, and every participant was able to complete all protocols without any issues, supporting acceptability of the experiment. Steps could be taken to further increase comfort, such as adding rubber pads to the forehead optodes.

It has been documented that DT walking generally results in PFC activation in healthy adults (Vitorio et al., 2017). Results for protocol 1 (Figure 5a–c) where we observed an increase in HbO during DT in channel AF8‐F6, roughly corresponding to BA46/dorsolateral PFC (dlPFC), are comparable to Fraser et al. (2016). Their study found significant increases in HbO levels in the dlPFC during DT walking with an n‐back task for both older and young adults. The dlPFC is associated with executive functions (Yogev‐Seligmann et al., 2008), which could indicate the increased need for executive resources during DT. While protocol 2 elicited no significant increase in HbO, protocol 3 (Figure 5f–g) led to increases in medial brain regions (roughly BA8), which could indicate that the navigational DT requires different resources. These results also support the scientific feasibility of distinguishing between DT and ST conditions. Contrasts between active conditions were reinforced by considering both HbO and HHb, indicating the importance of combined measures, which has been repeatedly argued for (Hakim et al., 2022).

A novelty of this feasibility study is to measure activation in PFC during three different complex walking protocols, not limiting experiments to steady‐state straight walking. The complex walking did not introduce lower SCI values than straight walking (Table 2). Normal walking, a relatively simple motor task, has been documented to not induce much blood oxygenation in the PFC, at least in young healthy adults (Mirelman et al., 2014). While straight walking (Figure 5b and d) did not elicit significant increase compared to baseline, neither did navigated walking in protocol 2 (Figure 5e). This could suggest that our navigation task might not have been challenging enough for a healthy population. However, navigation caused a significant HbO increase in protocol 3 (Figure 5f) but not in protocol 2 (Figure 5e). The reasons for this need further investigation.

### 4.1 Limitations

Although the sample size was sufficient to determine feasibility of fNIRS to measure PFC during complex walking, it might be too small to draw any neurophysiological conclusions. The sample was also highly physically active, something which might not be true for other populations, for example, neurodegenerative disease. It has been documented that oxygenation levels during DT walking can be affected by age (Mirelman et al., 2014); therefore, a limitation of our study could be the large variability in age (18–64 years) in the present study sample. Our future studies will separate these groups with more participants in each.

To account for extracerebellar blood flow, we included short channels. Nevertheless, it is not completely possible to disentangle cerebral and extracerebral signal components since we did not measure blood pressure or muscle movement. The signal could be biased by jaw movements during auditory Stroop and the oxygenation drop due to exhalation (Scholkmann et al., 2013). Finally, exact anatomical registration of optodes was not performed, for example, with structured‐light 3D scanning (Homölle & Oostenveld, 2019), which could make comparisons between subjects in our study less precise.

## 5 CONCLUSION

Our study supports both the process and scientific feasibility of fNIRS measurement of PFC activity during complex walking. The measurement systems were deemed acceptable by the participants, and adherence to the protocols was maintained. The complex walking tasks elicited differential brain activity in healthy participants and signal quality was high. This underlines the scientific feasibility of our setup, the usability of fNIRS, and can further be used for development of walking protocols, enabling reproducibility and a common test protocol when using this type of complex walking task.

## CONFLICT OF INTEREST STATEMENT

The authors declare no conflicts of interest.

## FUNDING

This study was supported by grants Swedish Parkinson Foundation (1327/21); Promobilia Foundation (21012); Norrbacka‐Eugenia foundation (N/A); Center for Innovative Medicine, Karolinska Institutet (FoUI‐973826); Doctoral School in Health Science, Karolinska Institutet (N/A); and Swedish state under the agreement between the Swedish government and the county councils, the ALF‐agreement (FoUI‐973200).

### ETHICS STATEMENT

This study was approved by the Regional Board of Ethics in Stockholm (Dnr 2020–05315 and 2021‐01329).

### PEER REVIEW

The peer review history for this article is available at https://publons.com/publon/10.1002/brb3.2948.

## PATIENT CONSENT STATEMENT

All participants received verbal and written information and gave written consent prior to study participation.

## Supporting information

Appendices

## Data Availability

Code is available via: https://osf.io/f4p3d/?view_only = c339fe6a5fce44408765a40788b06763. With respect to the Swedish and EU personal data legislation (GDPR), the data are not freely accessible due to regulations regarding personal integrity in research, public access and privacy. The data are available from the principal investigator of the project: Erika Franzén (erika.franzen@ki.se), on a reasonable request. Any sharing of data will be regulated via a data transfer and user agreement with the recipient.
