# Supplementary material for: Using functional near‐infrared spectroscopy to measure prefrontal cortex activity during dual‐task walking and navigated walking: A feasibility study
Source: Brain Behav. 2023 Mar 14;13(4):e2948. doi: 10.1002/brb3.2948 (PMC10097069; doi:10.1002/brb3.2948)
Supplement: Supplementary file 1 — Appendices [file BRB3-13-e2948-s001.docx]

| **Appendix A, feasibility questionnaire (Original Swedish version)**  **Följande frågor handlar om upplevelsen under eller efter gångtestet** | | | | | |
| --- | --- | --- | --- | --- | --- |
|  | Stämmer inte alls | Stämmer ganska dåligt | Stämmer ganska bra | Stämmer helt | Vet ej |
| 1. Jag upplevde att utrustningen var komfortabel att bära | 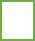 | 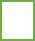 | 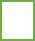 | 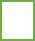 | 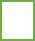 |
| 1. Jag upplevde att testningen tog för lång tid | 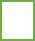 | 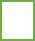 | 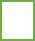 | 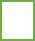 | 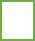 |
| 1. Jag upplevde trötthet under mätningen | 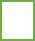 | 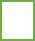 | 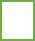 | 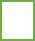 | 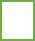 |
| 1. Jag upplevde trötthet efter mätningen | 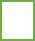 | 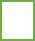 | 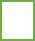 | 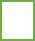 | 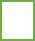 |
| 1. Jag upplevde smärta under mätningen   Var?................................. | 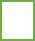 | 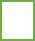 | 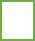 | 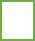 | 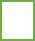 |
| 1. Jag upplevde smärta efter mätningen   Var?................................. | 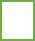 | 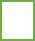 | 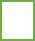 | 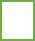 | 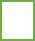 |
| 1. Jag upplevde yrsel under mätningen | 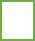 | 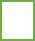 | 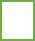 | 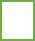 | 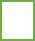 |
| 1. Jag upplevde yrsel efter mätningen | 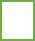 | 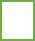 | 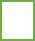 | 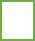 | 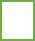 |
| 1. Jag var koncentrerad under uppgifterna | 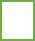 | 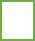 | 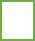 | 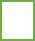 | 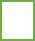 |
| **Följande frågor gäller uppgiften då du gick rakt framåt och lyssnade på orden hög och låg i hörlurarna.** | | | | | |
|  | Stämmer inte alls | Stämmer ganska dåligt | Stämmer ganska bra | Stämmer helt | Vet ej |
| 1. Jag upplevde att stå och säga hög/låg var svårt/utmanande | 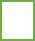 | 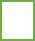 | 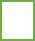 | 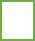 | 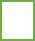 |
| 1. Jag upplevde att gå framåt och säga hög/låg var svårt/utmanande | 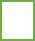 | 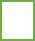 | 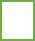 | 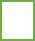 | 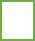 |
| 1. Jag upplevde att jag fokuserade mer på en utav uppgifterna | Ja, att gå framåt 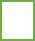 Ja, att svara 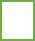 Nej 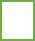 | | | | |
| 1. Jag upplevde att en av uppgifterna var viktigare än den andra | Ja, att gå framåt 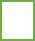 Ja, att svara 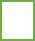 Nej 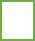 | | | | |
| **Följande frågor gäller uppgiften då du svängde mellan koner (navigerade) och lyssnade på orden hög och låg i hörlurarna.** | | | | | |
|  | Stämmer inte alls | Stämmer ganska dåligt | Stämmer ganska bra | Stämmer helt | Vet ej |
| 1. Jag upplevde att gå och svänga runt konerna var svårt/utmanande | 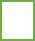 | 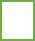 | 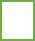 | 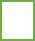 | 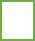 |
| 1. Jag upplevde att gå och svänga runt konerna och samtidigt säga hög/låg var svårt/utmanande | 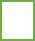 | 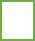 | 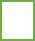 | 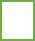 | 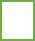 |
| 1. Jag upplevde att jag fokuserade mer på en utav uppgifterna | Ja, att navigera 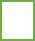 Ja, att svara 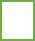 Nej 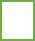 | | | | |
| 1. Jag upplevde att en av uppgifterna var viktigare än den andra | Ja, att navigera 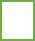 Ja, att svara 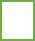 Nej 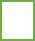 | | | | |

| **Appendix B, feasibility questionnaire (Translated English version)**  **The following questions pertains to the experience during or after the gait test** | | | | | |
| --- | --- | --- | --- | --- | --- |
|  | Strongly disagree | Disagree | Agree | Strongly agree | Don’t know |
| 1. The equipment was comfortable to wear | 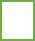 | 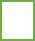 | 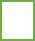 | 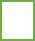 | 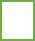 |
| 1. The gait test was too time consuming | 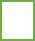 | 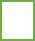 | 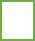 | 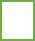 | 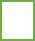 |
| 1. I experienced fatigue during the gait test | 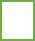 | 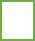 | 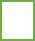 | 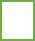 | 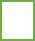 |
| 1. I experienced fatigue after the gait test | 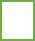 | 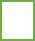 | 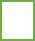 | 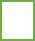 | 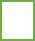 |
| 1. I experienced pain during the gait test   Where?................................. | 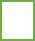 | 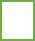 | 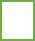 |  |  |
| 1. I experienced pain after the gait test   Where?................................. |  |  |  |  |  |
| 1. I experienced dizziness during the gait test |  |  |  |  |  |
| 1. I experienced dizziness after the gait test |  |  |  |  |  |
| 1. I was concentrated during the tasks |  |  |  |  |  |
| **The following questions pertains to the task when you walked straight and listened to the words high or low in the headphones.** | | | | | |
|  | Strongly disagree | Disagree | Agree | Strongly agree | Don’t know |
| 1. I experienced that standing still and answering high or low was difficult/challenging |  |  |  |  |  |
| 1. I experienced that walking while answering high or low was difficult/challenging |  |  |  |  |  |
| 1. I felt that I focused more on one of the tasks | Yes, walking Yes, answering No | | | | |
| 1. I felt that one of the tasks was more important than the other | Yes, walking Yes, answering No | | | | |
| **The following questions pertain to the task when you navigated and listened to the words high or low in the headphones.** | | | | | |
|  | Strongly disagree | Disagree | Agree | Strongly agree | Don’t know |
| 1. I experienced that navigating was difficult/challenging |  |  |  |  |  |
| 1. I experienced that navigating while answering high or low was difficult/challenging |  |  |  |  |  |
| 1. I felt that I focused more on one of the tasks | Yes, navigating Yes, answering No | | | | |
| 1. I felt that one of the tasks was more important than the other | Yes, navigating Yes, answering No | | | | |

**Appendix C, additional tables**

Table C.1 shows the average beta coefficients (β) after GLM analysis (average amplitudes of changes in HbO and HHb) for each channel pairing and participant over the different task conditions. Most tasks had a relatively higher concentration change of HbO compared to baseline with navigation (β=1.75) and dual-task navigation (β=1.54) having the greatest effect on brain oxygenation on average.

| **Table C.1:** Average beta coefficient value after GLM analysis over all the source and detector pairs for all participants for different conditions during the three protocols. | | |
| --- | --- | --- |
| *Protocol 1* | HbO | HHb |
| Standing ST, mean (SD) | 0.48 (3.31) | 0.06 (1.95) |
| Walking ST, mean (SD) | 0.86 (6.19) | 0.49 (3.04) |
| Walking DT, mean (SD) | 0.41 (6.27) | -0.13 (3.02) |
| *Protocol 2* |  |  |
| Walking ST, mean (SD) | 0.94 (7.97) | 0.30 (3.79) |
| Navigation ST, mean (SD) | 1.75 (9.52) | 0.47 (4.65) |
| *Protocol 3* |  |  |
| Navigation ST, mean (SD) | 1.55 (10.71) | 0.74 (4.94) |
| Navigation DT, mean (SD) | 1.54 (10.22) | 0.37 (4.93) |

Table C.2 shows oxygenated (HbO), deoxygenated (HHb), and total (HbT) hemoglobin concentration changes per condition (μM), averaged across all participants and source and detector pairs (channels) excluding short-separation channels, obtained after applying the modified Beer-Lambert Law. Navigated walking had the highest average change in HbT concentration (21.55 μM).

| **Table C.2:** Average hemoglobin concentration changes (μM) over all the source and detector pairs for all participants for different conditions during the three protocols. | | | |
| --- | --- | --- | --- |
| *Protocol 1* | HbO | HHb | HbT |
| Standing ST, mean (SD) | 0.15 (36.1) | 2.11 (23.19) | 2.26 (49.57) |
| Walking ST, mean (SD) | 7.52 (43.64) | 0.42 (25.69) | 7.57 (57.11) |
| Walking DT, mean (SD) | 8.04 (39.52) | -1.02 (23.09) | 7.02 (52.49) |
| *Protocol 2* |  |  |  |
| Walking ST, mean (SD) | -3.01 (39.39) | 0.02 (17.12) | -2.99 (45.43) |
| Navigation ST, mean (SD) | 20.69 (44.36) | 0.85 (17.75) | 21.55 (51.93) |
| *Protocol 3* |  |  |  |
| Navigation ST, mean (SD) | 14.25 (48.39) | 1.63 (23.52) | 15.89 (63.93) |
| Navigation DT, mean (SD) | 14.74 (41.74) | 0.98 (22.38) | 15.72 (55.00) |

Table C.3 shows an ROI analysis of conditions contrasted against each other for HbO, HHB and HbDiff (HbO – HHb). HbDiff values were obtained after adding HbDiff to the 1^st^ and 2^nd^ level analysis and re-running the ROI analysis.

The results for protocol 1 showed a trend of increase in HbO and decrease in HHb for ST and DT walk compared to standing, and DT walk compared to ST walk, although only the HHb decrease was significant for DT walk conditions compared to others. A similar non-significant difference was found in protocol 2 comparing navigation to ST walk, while both measures decreased in protocol 3 comparing DT navigation to ST navigation.

Significant differences were found comparing HbDiff, with a significant increase in protocol 1 for DT walk vs standing ST and a large although nonsignificant increase in DT walk vs ST walk, while the smallest increase was observed in ST walk vs standing ST. For protocol 2, HbDiff showed significant increase during navigated walking compared to straight walking, while no significant difference was found in protocol 3.

| **Table C.3:** ROI analysis of active conditions contrasted against each other.  Significant contrasts (q < 0.05) are marked in bold. | | | |
| --- | --- | --- | --- |
| *Protocol 1* | HbO | HHb | HbDiff |
| Walking DT - Standing ST, T (q) | 1.800 (0.078) | **-3.875 (0.001)** | **2.652 (0.012)** |
| Walking DT – Walking ST, T (q) | 0.441 (0.662) | **-3.463 (0.002)** | 1.903 (0.095) |
| Walking ST – Standing ST, T (q) | 1.387 (0.344) | -0.298 (0.767) | 0.722 (0.474) |
| *Protocol 2* |  |  |  |
| Navigation ST - Walking ST, T (q) | 1.220 (0.231) | -2.013 (0.104) | **3.159 (0.010)** |
| *Protocol 3* |  |  |  |
| Navigation DT – Navigation ST, T (q) | -0.711 (0.483) | -1.338 (0.381) | -0.585 (0.563) |

Table C.4 shows average peak spectral power values (26) obtained from the power spectrum of cross-correlated signals of each NIRS wavelength, calculated in MATLAB. The peak power is a complement to SCI and reflects sporadic movement artifacts. A threshold for a good-quality channel is 0.1. Navigation DT had the lowest peak power, while Standing ST had the highest peak power.

| **Table C.4:** Average peak spectral power values over all source and detector pairs for all participants for different protocol conditions. | |
| --- | --- |
| *Protocol 1* | Power, mean (SD) |
| Standing ST (Standing still while preforming AS) | 0.230 (0.142) |
| Walking ST (Straight walking) | 0.183 (0.110) |
| Walking DT (Straight walking while preforming AS) | 0.175 (0.109) |
| *Protocol 2* |  |
| Walking ST (Straight walking) | 0.184 (0.111) |
| Navigation ST (Navigated walking) | 0.161 (0.108) |
| *Protocol 3* |  |
| Navigation ST (Navigated walking) | 0.160 (0.108) |
| Navigation DT (Navigated walking while performing AS) | 0.147 (0.105) |
